# The estimated prevalence of osteoporosis in Bahrain: a multi-centered-based study

**DOI:** 10.1186/s12891-023-07145-8

**Published:** 2024-01-02

**Authors:** Adla Bakri Hassan, Yasin I. Tayem, Mir Sadat-Ali, Amer J. Almarabheh, Abdulhameed Alawadhi, Ahsan J. Butt, Haitham Jahrami, Jamal Saleh, Mai E. Matar, Mansoor Shaikh, Salman k. Hasan, Ali R. Karashi

**Affiliations:** 1 College of Medicine and Medical Sciences, Arabian Gulf University, Manama, Kingdom of Bahrain; 2 King Abdullah Medical City, Manama, Kingdom of Bahrain; 3 Salmaniya Medical Complex, Government Hospitals, Manama, Kingdom of Bahrain; 4 King Hamad University Hospital, Manama, Kingdom of Bahrain; 5 Psychiatric Hospital, Government Hospitals, Manama, Kingdom of Bahrain; 6 Orthocare, Orthopaedic Centre, Manama, Kingdom of Bahrain

**Keywords:** Bone Mineral Density, Osteopenia, Bahraini, DEXA, Menopause

## Abstract

**Objectives:**

the primary aim of this study was to examine the prevalence and risk factors of low bone mineral density in Bahrain.

**Methods:**

this was a retrospective study, which targeted a cohort of 4822 Bahraini subjects (mean age 59.36 years: 93% females). Demographic data and results of lumbar and femur DEXA scan for the targeted sample, over the period 2016–2018, were retrieved from four hospitals.

**Results:**

The prevalence of low BMD was 62.3% (46.4% had osteopenia and 15.9% had osteoporosis). The highest rate of osteopenia was detected at the age group younger than 44 years. However, with increasing age, the rate of osteopenia declined, whereas osteoporosis increased (P < 0.001). Females were found to be at higher risk of developing both osteopenia (45.8%) and osteoporosis (18.1%) compared to males (39% and 12.4%, respectively) (P < 0.001). Postmenopausal women exhibited higher rates of low BMD (42.4% osteopenia, 22.3% osteoporosis) compared to elderly men (30.9% osteopenia, 9% osteoporosis).

**Conclusions:**

We reported high prevalence of osteopenia and osteoporosis in Bahrain. Low BMD was more common in females, especially in postmenopausal women. Highest prevalence of osteopenia happened at young age. Therefore, we advocate screening at younger age than previously recommended.

## Introduction

Osteoporosis is a silent bone disease characterized by the development of brittle bones and is associated with an increased risk of catastrophic hip and spine fractures [1]. The progressive loss of bone mineral density (BMD) in osteoporosis, not only results from reduced bone mass, but also due to ongoing deterioration of bone microarchitecture [2]. Osteoporosis, and its consequent fractures, is primarily a disease of postmenopausal women, albeit older men are not immune [3], as well as people with other comorbidities [4]. It results in significant morbidity, mortality, and economic burden, primarily because of the disastrous hip and spine fractures [5, 6]. Clinically, the diagnosis of osteoporosis is made when BMD is 2.5 standard deviations or more below the mean of young healthy adult (T-score ≤ − 2.5), and relative to same age and gender for men or young subjects (Z score <-2.5) [7]. Despite the increase in the availability of a wide range of effective medications for the prevention and treatment of osteoporosis, this disease is largely considered under-treated, especially in older patients and those with comorbidities [8].

Although several risk factors underlie the development of osteoporosis, increasing age and female gender are the two most important [9]. Indeed, osteoporosis is frequently described as a disease of postmenopausal women because it is common in this group [10]. The reason for bone loss in women after menopause is primarily related to the low estrogen levels during this period in women’s life [11, 12]. Similarly, females who undergo pre-menopausal oophorectomy and those who suffer from premature ovarian failure, present with pre-mature osteoporosis [12, 13]. The body mass index (BMI) is another determinant for developing osteoporosis because following menopause estrogen is produced within the adipose tissue from its androgen precursor. This explains the lower risk of osteoporosis in obese postmenopausal women [12, 13]. Dietary factors are also believed to play a role in the causation of osteoporosis, particularly low intake of calcium and vitamin D, however, whether calcium and vitamin D supplement alone preserves bone mass is yet to be determined [14]. Certain lifestyle factors also predispose to osteoporosis, including lack of physical activity, alcohol intake, and cigarette smoking [5]. Chronic therapy with glucocorticoids is strongly associated with loss of BMD, and patients who need long-term corticosteroids must be monitored for the development of osteoporosis [15].

Globally, it is estimated that around one-third of women and one-fifth of men suffer from an osteoporotic fracture during their lifetime [5]. The prevalence of osteoporosis differs between countries, but as the prevalence of longevity prevails, the risk of osteoporosis and catastrophic fractures increases [16–18]. In general, the prevalence of this metabolic bone disease remains higher in the Western countries, compared to the rest of the world. Indeed, a recent report on the prevalence of fragility fractures in six European countries (Sweden, France, Germany, Italy, Spain and the UK) revealed that the number of patients who are expected to develop low BMD is expected to escalate from 2.7 million to 3.3 million in 2030 [19]. The direct and indirect societal and economic costs of osteoporotic fractures are huge. Specifically, a hip fracture, which is the most severe and catastrophic complication of osteoporosis, results in the costliest hospitalization, long-term care, impaired quality of life, disability, and death [19]. In 2017, the National Institute for Health and Care Excellence (NICE) introduced guidelines, which stated that only two groups of postmenopausal women should be treated with medications for osteoporosis based on the presence or absence of history of fracture as secondary and primary prevention, respectively [20]. Although in the EMR, osteoporosis is considered a major public health problem [21], the data on the rate of osteoporosis in Bahrain is scarce. To our knowledge, there has been no nationwide studies, which investigated the prevalence of this health problem in Bahrain.

The main objectives of this study were to determine the prevalence of osteoporosis in Bahrain and to identify the risk factors that predispose to low BMD such as age and gender. Moreover, we ought to update the recommendations on the most appropriate age of BMD screening.

## Materials and methods

### Ethical approval

Prior to the collection of data, this study was approved by the Research and Ethics Committee at the Arabian Gulf University (number: E003-PI-10/18). In addition, we also received approval from the Secondary Health Care Research Sub Committee at Salmaniya Medical Complex, Orthocare and King Hamad University Hospital (number 273/2019).

### Settings

#### Participants and study design

This was a retrospective study, which used universal sampling to select participants. That is, all subjects who visited the radiology departments for DEXA scan and were evaluated for their BMD results in the four target hospitals during the selected study period were included in this study. Data were collected from four hospitals in Bahrain: Salmaniya Medical Complex (SMC), King Abdallah Medical City (KAMC), King Hamad University Hospital (KHUH), and Orthocare center. Bahrain has seven public hospitals and 18 private ones, with the number of available hospital beds standing at 2107 in the public sector versus 457 in the private sector.

SMC is the main public hospital, and the largest tertiary hospital in Bahrain, which also functions as a teaching and research centre for Health Professionals. Established in 1957 and having a bed capacity of approximately 1,200 beds. KHUH was recently established in 2010 as a big public hospital. It also functions as a university teaching hospital and research Centre for Health Professionals and accommodates around 410 beds. KAMC is a public hospital, with multi-clinics established in 2011, currently with around 100 beds and plan to accommodates more than 300 beds. Orthocare Centre is a private hospital, and one of the most specialized orthopedic clinics in the Middle East.

Data were collected over three years: January 2016 to December 2018. Due to the retrospective nature of the study and difficulty to get data directly from the participants about their menopausal status, postmenopausal women were based on being more than 56 years of age, based on previous local studies by our research group. Demographic data of participants including age and gender were collected by retrieving the information from the electronic medical records of patients in the target hospitals.

#### Bone mineral density (BMD) measurement

Collected data also included bone densitometry dual-energy x-ray absorptiometry (DEXA). The machine used for performing DEXA scan was General Electric Lunar, Chicago, Illinois, USA and the software used was the GE NHANES III software update. BMD was expressed as the total bone mineral content (g) divided by the surface area (cm^2^). BMD was measured for both the left femur neck and the lumbar spine for all the subjects. All BMD measurements were carried out by trained technicians. Those technicians performed daily calibration of the employed machine as per the manufacturer`s instructions. The interpretation of the BMD measurements was based on the World Health Organization (WHO) criteria. That is, osteoporosis was diagnosed if the T score was − 2.5 or below (≤ 2.5), whereas osteopenia was diagnosed when the T score was between − 1.0 and − 2.5 [7]. When discrepancy was found between lumbar and femur BMD, the lowest score was adopted, and based on that score the subjects were categorized into normal, osteopenia or osteoporosis.

Considering the scope and objectives of our research, which focused on assessing the prevalence of osteopenia using a widely accepted and accessible method, EDXA was the most appropriate choice. It allowed us to efficiently collect data on BMD in a large cohort of participants and obtain reliable measurements for our analysis.

### Statistical analysis

The data collected in this study was analyzed by using the Statistical Package for the Social Sciences (SPSS), Version 27 (IBM Corp., Chicago, Illinois, USA). Arithmetic mean ± standard deviation was calculated for continuous variables, whereas proportions were calculated for categorical variables. A cluster bar graph was plotted to represent the distribution of the two qualitative variables, whereas a line diagram was used to represent the mean of quantitative variables. An independent sample t-test was used to compare the results between groups, and the Chi-Square test was employed to compare the proportions between categorical variables. A *p*-value of less than 0.05 was considered statistically significant.

## Results

The demographic data of the participants in this study are represented in Table 1. The total number of Bahraini subjects who were included in this investigation was 4822. The mean age of participants was 59.36 years (SD = 11.19). Most subjects were aged 44–75 (82.8%). The remaining subjects were aged less than 44 (8.7%), or more than 75 (8.5%). According to gender, majority of subjects were females (92.9%), whereas the rest were males (7.1%). The number of cases according to year of testing was almost comparable. According to the source of data, 71.8% of the subjects were from SMC, 20.6% were from KHUH, 6.5% were from KAMC and 1.1% were obtained from Orthocare.

**Table 1:** Demographic data of the participants (n = 4822)

Characteristic	Subgroup	No	%
**Age / year**			
**Mean (SD)**	**59.36 ± 11.19**		
Age category	< 44	414	8.7
	44 - < 60	2016	42.0
	60 - < 75	1921	40.8
	> 75	411	8.5
Year of testing	2016	1163	33.6
	2017	1123	32.4
	2018	1180	34.0
Gender	Male	328	7.1
	Female	4285	92.9
Hospital	SMC	3466	71.9
	KHUH KAMC	992 312	20.6 6.5
	Orthocare	52	1.1

SMC = Salmaniya Medical Complex; KHUH = King Hamad University; KAMC = King Abdullah Medical Center

We studied the total BMD (combined femur and lumbar spine) based on age group (Table 2). Our data showed that 62.3% of our sample had low BMD (46.4% had osteopenia, 15.9% had osteoporosis). When the data were segregated based on age, the rate of osteopenia progressively declined from 69.3% at the age below 44 to 37.4% at the age group beyond 75. In contrast, the prevalence of osteoporosis followed the opposite trend. That is, osteoporosis increased from 7.2% at the youngest age group to 27.3% at the eldest age category. A statistically significant difference was revealed between the age groups (P < 0.001). When the data were analyzed based on gender, females were found to be at higher risk of developing both osteopenia (45.8%) and osteoporosis (18.1%) compared to males (39% and 12.4%, respectively) (P < 0.001). Similarly, when the data were compared between elderly men and postmenopausal women, we found that the latter exhibited higher rates of low BMD. That is, 42.4% had osteopenia while 22.3% had osteoporosis. In elderly men, however, osteopenia was detected in 30.9% whereas; osteoporosis was seen in 9% (data not shown). The difference between the means was significant in postmenopausal women age groups (P < 0.005), but not in elderly men (Table 2).

**Table 2:** Total BMD category according to age and gender

	BMD				
	**Normal** n = 1678 (37.7%)	**Osteopenia** n = 2237 (46.4%)	**Osteoporosis** n = 767 (15.9%)	**Total** (4682)	***p. value***
**Age group**					
< 44	91 (23.5)	269 (69.3)	28 (7.2)	388 (100)	< 0.001
44-<60	734 (36.9)	1019(51)	237 (11.9)	1990 (100)	
60-<75	710 (37.4)	797 (42)	391 (20.6)	1898 (100)	
≥ 75	143 (35.2)	152 (37.4)	111 (27.3)	406 (100)	
**Postmenopausal women**					
56 - <60 60-<75	229 (35.8) 630 (35.8)	280 (43.8) 752 (42.8)	131 (20.5) 376 (21.4)	640 (100) 1758 (100)	< 0.005
≥ 75	116 (31.7)	140 (38.3)	110 (30.1)	366 (100)	
**Elderly men**					0.235
60-<75	80 (58)	43 (31.2)	15 (10.9)	138 (100)	
≥ 75	27 (67.5)	12 (30)	1 (2.5)	40 (100)	
**Gender**					
Male	153 (48.6)	123 (39)	39 (12.4)	315 (100)	< 0.001
Female	1522 (36.1)	1930(45)	762 (18.1)	4214 (100)	

BMD = bone mineral density

Table 3 revealed our results based on the lumbar spine BMD, the participants were found to have normal BMD (1846, 40%), osteopenia (2054, 44.5%) or osteoporosis (713, 15.5%). Subjects younger than 44 had high prevalence of osteopenia (66.8%). However, BMD values progressively increased for the older age groups, and were highest in those who were 75 years or older. In contrast to those observations, the rate of osteoporosis followed an opposite trend compared to that of osteopenia. That is, BMD values were lowest in those who were aged more than 75 years and highest at the young age groups. The differences between the means of those groups were significant (*P* value < 0.001). When the lumbar spine BMD measurements were compared based on gender, females had higher prevalence of osteopenia and osteoporosis. Moreover, the rate of osteopenia in both males and females (35.2% and 42.5%, respectively) was significantly higher than osteoporosis (11.9% and 17.0%, respectively). Chi-square analysis revealed a significant difference in means between males and females (p < 0.001).

**Table 3:** Lumbar spine BMD according to age and gender

	Lumbar spine				
	**Normal** n = 1846 (40%)	**Osteopenia** n = 2054 (44.5%)	**Osteoporosis** n = 713 (15.5%)	**Total** (4613)	***p. value***
**Age group**					
< 44	107 (27.1)	264 (66.8)	24 (6.1)	395 (100)	< 0.001
44-<60	788 (39.9)	962 (48.7)	227 (11.5)	1977 (100)	
60-<75	784 (42.4)	695 (37.6)	368 (19.9)	1847 (100)	
≥ 75	167 (42.4)	133 (33.8)	94 (23.9)	394 (100)	
**Gender**					
Male	164 (52.9)	109 (35.2)	37 (11.9)	310 (100)	< 0.001
Female	1683 (40.5)	1763 (42.5)	707 (17)	4153 (100)	

BMD = bone mineral density

Our initial results indicating that individuals with lower BMD levels, particularly those diagnosed with Osteoporosis, are more likely to experience Fragility fractures. Among the 430 fractures in total 45.12% (194) were fragility fractures (osteoporosis‑related fractures).

Patients were also grouped based on femur BMD results (Table 4). Overall, our results indicated that 3192 (68.4%) subjects had normal BMD, 1239 (26.6%) had osteopenia and 236 (5%) had osteoporosis. Our data revealed that the rate of osteopenia was lowest in subjects who were below the age of 44 (13.1%) but increased progressively with age and reached 33.3% in those beyond 75 years. Generally, the same trend was observed for the prevalence of osteoporosis, where the rate increased from 3.9% at the younger age group to 17.9% for the eldest one. Chi square showed a significant difference in means of the age groups (p < 0.001). Based on gender, females were at higher risk of osteoporosis compared to males (5.8% and 3.2%, respectively), as well as osteopenia (27.9% and 23.3%, respectively) and the difference was statistically significant (p < 0.05).

**Table 4:** Femur BMD according to age and gender

	Femur				
	**Normal** (n = 3192, 68.4%)	**Osteopenia** (n = 1239, 26.6%)	**Osteoporosis** (n = 236, 5%)	**Total** (4667)	***p. value***
**Age group**					
< 44	322 (83)	51 (13.1)	15 (3.9)	388 (100)	< 0.001
44-<60	1509 (76)	438 (22.1)	39 (2)	1986 (100)	
60-<75	1164 (61.6)	616 (32.6)	110 (5.8)	1890 (100)	
≥ 75	197 (48.9)	134 (33.3)	72 (17.9)	403 (100)	
**Gender**					
Male	230 (73.5)	73 (23.3)	10 (3.2)	313 (100)	< 0.05
Female	2790 (66.3)	1173 (27.9)	243 (5.8)	4206 (100)	

BMD = bone mineral density

The co-morbidities between the participants reported as follow: endocrinological diseases were 71% (3434 patients) among them diabetes mellitus (DMT2) was 63.6% (2183), followed by rheumatological diseases 39.8% (1920 patients) and among them rheumatoid arthritis was 14.7% (283), and malignancy 19.4% (941 patients) and among them breast cancer was 75.8% (713). Additionally, the most reported medications used by the patients as reported in their electronic files were: vitamin D, Calcium, Methotrexate, Steroids, Denosumab, and Bisphosphonates.

## Discussion

Osteoporosis is an underdiagnosed public health problem in many countries, largely because it is asymptomatic in most cases until serious sequela take place [22]. The most serious complications of osteoporosis are catastrophic fractures of the spine and hip, which are widely perceived as the most costly health burdens on both the individual and society [23]. This study aimed to investigate retrospectively the risk factors and prevalence of osteoporosis and osteopenia in Bahrain by targeting a cohort of 4822 Bahraini subjects in four hospitals over a period of three years. Our data revealed a high prevalence of both osteoporosis and osteopenia, but very high rate of osteopenia was detected at young age groups compared to osteoporosis. Our results also showed that low BMD increased with age and was more common in females, postmenopausal women, and elderly.

Our study revealed that the overall prevalence of low BMD was relatively high and reached 62.3%, with more subjects having osteopenia (46.4%) than osteoporosis (15.9%). The high prevalence of osteopenia in our sample is very scary and unexpected. A previous study showed that 23.7% of patients with osteopenia progressed to osteoporosis with a median progression time of more than 8.5 years and 3.2 years in low risk and high-risk group, respectively. That study recommended the BMD testing interval should be 1–2 years [24].

The data on the prevalence of low BMD in Bahrain is scarce. Indeed, the first study in Bahrain, which was published in 2020, was conducted over two year’s period, in a single center, with a relatively small number of patients (251 patients) [25]. That study revealed that the prevalence of osteoporosis was 38.5%, which was significantly higher than what we reported in our current study. We can say that the sensitivity of our study, by examining more patients (312 patients), large duration (3 years) from same the center, and including additional more three centers, is more than the sensitivity of the study by Sadat-Ali [25]. The second study published in 2021, aimed to estimate the prevalence of osteoporosis among young women (47 +/- 11 years) attending the health centers in Bahrain. The study comprised of 892 female subjects, and revealed that there was 66% had normal BMD, 29% had osteopenia, and 4.9% had osteoporosis [26]. While that study examined specific age group in only female gender, our study examined all age groups in both genders. The prevalence of osteoporosis in our sample was also lower than many of the regional countries, but comparable with Kuwait (15.1%) country [21]. A systematic review and meta-analysis were conducted on the prevalence of osteoporosis in the EMR for the period 2000–2017. It was reported that the overall prevalence of osteoporosis was 24.4% (31,593 participants). The prevalence increased significantly over the study years, from 19.8 to 32.7% thereafter. The highest prevalence was in Saudi Arabia (32.7%), whereas the lowest was in Kuwait (15.1%) [21]. However, Bahrain was not included in that study due to lack of studies over that period. Our results found no difference in the prevalence of osteoporosis among Bahrain’s populations over the three targeted years. This observation was not consistent with the expected increase in the prevalence of osteoporosis over the period of 2010 to 2020 with the increase in the ageing population [27].

Our data revealed that the prevalence of osteoporosis and osteopenia based on total BMD increased with age. That is, among those who were 60 years or older, 21.79% had osteoporosis. Those data are in line with previous evidence which showed a positive association between age and osteoporosis [28]. The prevalence of osteoporosis in our sample is comparable to the recently reported international rates. Indeed, a recent systematic review and meta-analysis on the global prevalence of osteoporosis, revealed that the overall rate of osteoporosis in the world population (age 15–105 years) reached 18.3% [29]. Another study estimated the prevalence of osteoporosis among age 50 years and older in several industrialized countries (USA, Cana da, five European countries, Australia, and Japan) using the WHO BMD-based definition of osteoporosis, reported the highest prevalence, more than 20%, were only from Japan (26.3%) and USA (21%) [30]. Multiple risk factors account for the increased prevalence of low BMD in older age groups. Those include vitamin D insufficiency, reduced Calcium absorption, inadequate exposure to sunlight, excessive smoking, and family history [31, 32]. The rate of osteopenia was highest at the youngest age group, then it declined with age. This decline in osteopenia with age might be explained by the possibility that subjects switched from osteopenia to osteoporosis. Another explanation is that this false increase in BMD could be related to the development of osteoarthritis that increases with age [33], which masked the low BMD due to subchondral sclerosis and osteophytes formation, despite, the fact that degenerative changes of the spine itself could be a cause of local BMD loss [29, 32]. The recommendations of the Saudi Osteoporosis Society state that all women above the age of 60 years must undergo BMD screening by using DEXA scan [34]. However, based on our data, which revealed early onset of osteopenia, we recommend screening at an early age in Bahrain.

The current study showed a higher prevalence of low BMD among females compared to males. Indeed, women were also found to have more osteopenia and osteoporosis compared to men. A previous study showed that women had a four times higher rate of osteoporosis and a two times higher rate of osteopenia compared to men [35]. Based on gender, the study of global prevalence of osteoporosis reported that, osteoporosis was more common in females compared to males (23.1% and 11.7%, respectively) [29]. Similar conclusion was reported in another recent systematic review and meta-analysis, which investigated the prevalence of osteoporosis in patients (3562 patients) undergoing total joint arthroplasty, and reported that the rate of osteoporosis in males, females, and postmenopausal females were 5.5%, 29.0%, and 38.3%, respectively [36]. Spinal and femur fractures are the most feared complications of osteoporosis. Those fractures constitute a huge economic and health burden on patients and health systems. The identification of women who have osteoporosis is of extreme importance to offer them aggressive pharmacological therapy to avoid the risk of imminent fractures. Unfortunately, even osteoporotic women who had history of fracture are frequently reported to be undertreated [37].

Our data revealed that postmenopausal women had higher prevalence of osteoporosis and osteopenia compared to elderly men. Those data are consistent with a previous study that investigated bone loss in peri- and postmenopausal women, who were followed for 16 years. The investigators reported that bone mass was progressively lost at menopause. In fact, women lost about 20% of their bone mass during the first 5 to 7 years following menopause. Moreover, it showed that premenopausal women with low age-specific BMD at the age of 48 years had an increased risk of experiencing low BMD at the age of 64 years [38]. Our results were supported by another study by Shilbayeh et al. who revealed that age at menopause had a strong independent association with decreased BMD in lumbar and femoral neck regions [18]. A French study estimated the future burden of postmenopausal osteoporosis in women aged ≥ 50 years. It predicted that the number of postmenopausal osteoporotic women was expected to increase from 3.0 million to 3.4 million between 2010 and 2020. The aging of populations is expected to drive a marked increase in the prevalence of osteoporosis and osteoporotic fractures. These expectations should guide future planning for providing appropriate health care for the population [27].

### Limitations of the study

Our study had certain limitations. Firstly, being a retrospective there was some missing data in the electronic records, thus, more than 2000 subjects were excluded from the first step in the analysis (original data were 6886). Secondly, no data was available on the measurement of wrist BMD, which should enhance the accuracy of the diagnosis of low BMD, however, as in many previous studies, we relied on the measurement of both lumbar and femur BMD. Thirdly, we could not rule out the potential bias of the data, as hospital-based data, most likely the BMD for many of those subjects were requested by their doctors for some reasons, as patients with co-morbidities were not excluded. Additionally, the medications used by those patients as reported in their electronic files could also affect the results.

We acknowledge that the use of DEXA Manufacturer’s reference data, particularly if based on US reference data, may not accurately reflect the specific characteristics and bone health of our study population. The reference data provided by the manufacturer serves as a standardized benchmark for comparison, but it may not fully capture the diversity and unique characteristics of our country’s population.

To address this limitation and obtain a more accurate assessment of osteopenia prevalence, we agree that future studies should consider developing country-specific reference data. A study specifically designed to establish reference values based on a representative sample of our population would provide more reliable and context-specific information.

On the other hand, our study has many strengths. Indeed, we included a large sample of subjects relative to the population of Bahrain. At the time of our study, the total population was less than 1.500,000 and the Bahrainis were around 600.000 only. Although our large sample is not representative, but this multicenter study provides valuable epidemiological data since there is no nationwide study investigating the prevalence of osteoporosis in Bahrain up to date.

## Conclusions

Our study revealed a high rate of osteopenia and osteoporosis in Bahrain. The major risk factors for the development of low BMD included female gender, especially postmenopausal women, and elderly age. A high prevalence of osteopenia was also detected at young age groups. Based on these data, to minimize the risk of catastrophic complications of osteoporosis, especially fractures of spine and femur, we recommend BMD screening at younger age groups of 44 years.

## Data Availability

Data are available upon request from the corresponding author (A.B.).
